# Developing useful early warning and prognostic scores for COVID-19

**DOI:** 10.1136/postgradmedj-2021-140086

**Published:** 2021-05-28

**Authors:** Charles Coughlan, Shati Rahman, Kate Honeyford, Céire E Costelloe

**Affiliations:** Global Digital Health Unit, Department of Primary Care and Public Health, Imperial College London, London, UK; Department of Tropical and Infectious Diseases, University College London Hospitals NHS Foundation Trust, London, UK; Global Digital Health Unit, Department of Primary Care and Public Health, Imperial College London, London, UK; Global Digital Health Unit, Department of Primary Care and Public Health, Imperial College London, London, UK; Global Digital Health Unit, Department of Primary Care and Public Health, Imperial College London, London, UK

## Introduction

Early warning or ‘track-and-trigger’ scores (EWSs) are used to identify the deteriorating patient and reduce unwarranted variation in the incidence of adverse events.^1^ They were developed to enable timely escalation of sick patients to medical staff and are used in everyday clinical practice to guide changes in clinical management, admission to intensive care units (ICUs) and initiation of end-of-life care. Early track-and-trigger scores were based on aggregate vital signs; many have been externally validated in hospital and prehospital settings as predictors of ICU admission and survival for sepsis,^2^ exacerbations of chronic obstructive pulmonary disease^3^ and trauma.^4^ Machine learning and the rollout of integrated electronic health records have accelerated the development of sophisticated EWSs incorporating blood test and imaging results. These scores may provide ‘real-time’ information about ongoing clinical deterioration or a more rounded overall assessment of prognosis. Some of these tools may improve outcomes in patients with life-threatening pathology,^5^ but others are methodologically flawed and may have no or even adverse effects on patient care.^1^

EWSs lose their salience when they fail to identify deteriorating patients and when staffing and resource limitations in overstretched healthcare systems prevent clinicians from taking timely action. The COVID-19 pandemic has placed immense pressure on health systems across the world, and adults with COVID-19 may deteriorate rapidly and unexpectedly.^6^ There is widespread concern that existing EWSs may underestimate illness severity in patients with COVID-19, providing clinicians with false reassurance and thus delaying treatment escalation.^7 8^ Several groups have therefore sought to assess the utility of existing track-and-trigger scores and develop and validate novel tools for adults with COVID-19. This article will outline the pitfalls of existing EWSs for adult patients with COVID-19, highlight key findings from studies of novel EWSs for COVID-19 and discuss the ideal properties of a track-and-trigger score for COVID-19 suitable for use around the world.

## What are EWSs and why are they useful in healthcare settings?

The first EWS emerged in the late 1990s. Early versions assigned numerical values to different vital signs, and other factors such as clinical intuition, with aggregate scores triggering escalation to medical staff. They were designed primarily to reduce the incidence of avoidable in-hospital cardiac arrests in ward settings by enabling timely transfer of sick patients to ICU. Scores were developed with poor methodological rigour and in a haphazard fashion with local and regional variations, until regulatory bodies and professional organisations pressed for and developed standardised tools. For example, in the UK, the Royal College of Physicians developed the National Early Warning Score (NEWS), which was launched in 2012 and soon became mandatory in National Health Service hospitals.^9^ To reflect differences in physiological norms, distinct EWSs have been developed for adult, paediatric and obstetric populations. In recent years, novel or adapted scores have focused on different outcomes, such as cause-specific or all-cause mortality, and have been designed for use in different settings (such as the emergency department (ED) and in primary and prehospital care).

There is some evidence that implementation of EWSs improves outcomes for patients with sepsis,^10^ and several studies support their utility in identifying critical illness in hospital and prehospital settings.^11 12^ EWSs also provide a common language for ‘sickness’ and aid triage and resource allocation, particularly in a pandemic setting. Nonetheless, frontline professionals are aware of their pitfalls, particularly for those scores based on physiological parameters. Isolated values must be interpreted with regard to trajectory and placed within a clinical context—junior doctors are often informed of a patient ‘triggering’ when they have had a high score for hours or even days and already been reviewed. EWS based on vital signs can also provide false reassurance; shocked patients on beta blockers may not mount a tachycardia, and patients with acute renal failure may show no respiratory, cardiovascular or neurological compromise despite requiring urgent renal replacement therapy.

## What are the problems with existing EWSs in relation to COVID-19?

Where clinically appropriate, the deteriorating patient with COVID-19 requires urgent clinical review to determine the need for non-invasive ventilation (NIV) or intubation and mechanical ventilation (IMV). Delays in accessing these time-critical interventions may result in adverse outcomes. Depending on the patient’s age, comorbidities, level of frailty and the nature of their acute illness, their ceiling of care may be limited to NIV or even ward-based treatment, in which case deterioration may represent a terminal event and prompt a switch to end-of-life care. Clinical signs of deterioration in hospitalised adults with COVID-19 include a rising oxygen requirement, raised respiratory rate, use of accessory muscles of respiration and altered mental state.

In NEWS2, the most widely used EWS in the UK, supplemental oxygen therapy scores two points, but once a patient is on oxygen this score does not change to reflect flow rate or oxygen delivery device. Work of breathing is not included in NEWS2, though it has been used as an inclusion criterion for NIV in COVID-19.^13^ NEWS2 was developed with a focus on sepsis and therefore assigns significant value to tachycardia and hypotension. However, cardiovascular compromise is relatively uncommon in moderate to severe COVID-19 and may indicate additional pathology such as bacterial sepsis or pulmonary embolism.^14^ While respiratory rate may rise as patients with COVID-19 deteriorate, there are widespread reports of ‘happy hypoxia’ in which the typical physiological response (tachypnoea and increased work of breathing) to and subjective experience of hypoxia (dyspnoea) are absent.^15 16^ A recent report suggesting that pulse oximetry monitoring may underestimate the frequency of hypoxaemia in black patients is of particular concern in the context of COVID-19.^17^

## Development of novel early warning and prognostic scores for COVID-19

Various research groups have investigated whether existing scores can accurately identify hospitalised patients with COVID-19 who are at risk of clinical deterioration. Several studies have suggested that EWSs such as NEWS2 and the quick Sequential (Sepsis-related) Organ Failure Assessment, and prognostic tools such as CURB-65 perform poorly in cohorts of inpatients with COVID-19.^18 19^ This has spurred the development of dozens of bespoke early warning and prognostic scores for COVID-19 through retrospective multivariable logistic regression of patient-level data.

While outcomes of interest and time horizons vary, most models have combined vital signs with demographic factors, comorbidities and laboratory and imaging indices which reflect risk factors for severe disease or death. Variables of interest have typically been identified by expert clinicians or derived from observational studies highlighting risk factors for adverse outcomes in early COVID-19 cohorts and for other respiratory illnesses such as bacterial pneumonia and influenza. Researchers have developed these composite scores by assigning differential weight to each variable and then evaluating the clinical sensitivity and specificity of candidate models at different thresholds for clinical deterioration. Scores favouring variables derived from the wisdom of frontline clinicians may be more tractable in clinical settings but may lack the discriminative power offered by data-driven scores based on statistical analysis of routinely collected patient-level data. Several groups have sought to balance these tensions by asking panels of clinicians to review the relevance of candidate variables identified by statistical analyses.

The trade-off between each model’s sensitivity and specificity can be represented by receiver operator characteristics (ROCs), which can be displayed graphically. By quantifying the ‘area under the ROC curve’ (AUROC) for new and existing models, it is possible to compare their performance. For existing and novel scores evaluated in COVID-19 cohorts, this could mean discrimination between stable and deteriorating hospitalised patients—where deterioration is defined by the subsequent need for IMV or ICU level care—or patients at high or low risk of mortality at first presentation to the ED. AUROC values always lie between 0 and 1; a value of 0.5 suggests that a model’s discrimination is no better than chance. We would consider an AUROC value over 0.75 to represent good clinical discrimination.^20^

As outcomes such as ICU admission and mortality are relatively rare events, models derived from small populations are at risk of ‘overfitting’; providing perfect results under study conditions but performing poorly in the real world. Some prognostic scores have combined the risk of SARS-CoV-2 exposure with the risk of severe COVID-19, despite differences in their respective risk factors. These risk prediction tools become less useful as exposures deviate from those seen in study conditions. This is particularly relevant to the issue of ethnic group differences in hospitalisation and mortality from COVID-19 in the UK and USA, which likely reflect differences in exposure to SARS-CoV-2 and confounding factors such as deprivation rather than any genetic differences in underlying risk profiles.^21^

Furthermore, most novel prognostic and EWSs for COVID-19 have been developed without prospective external validation in large and diverse patient cohorts. Unsurprisingly, a systematic review of prognostic scores for COVID-19 suggests that most novel scores are poorly reported and likely overestimate their true predictive performance.^22^ This is supported by a recent single-centre external validation study, which found that NEWS2 score was a better predictor of clinical deterioration at 24 hours than 22 novel prognostic scores in a cohort of 411 hospitalised adults with COVID-19, with an AUROC of 0.76.^23^ The sole high-quality novel scores with similar performance to NEWS2 after external validation are the Coronavirus Clinical Characterisation Consortium (4C) mortality (AUROC 0.78) and deterioration scores. Derived from multiethnic cohorts of over 30 000 hospitalised patients, these scores show real promise and have been widely adopted in the UK and beyond.

The 4C mortality score combines patient age; sex at birth; number of comorbidities; respiratory rate, peripheral oxygen saturations and Glasgow Coma Scale at admission; and serum urea and C reactive protein concentrations to provide an estimate of untreated in-hospital mortality.^24^ Patients receive an aggregate score out of 21, with age alone providing up to 8 points. By providing an early assessment of prognosis at the front door, the 4C score might be used to guide treatment decisions, triage and clinical disposition. However, it is important to note that it predicts mortality rather than the need for NIV, IMV or ICU admission. As such, it may be most useful at its extremes; giving clinicians confidence to discharge patients with low mortality scores or prompt early conversations around treatment escalation with older patients requiring oxygen. The 4C deterioration score incorporates 11 variables and defines clinical deterioration more broadly, to encompass death, ICU admission and IMV.^25^ It can be used at first presentation to ED for community-acquired COVID-19 or immediately after identification of nosocomial disease. This score may help to optimise resource allocation—for example, by prompting early transfer of high-risk patients to higher acuity settings—and inform discussions with patients and families to give them time to prepare for expected deterioration. Future studies should assess reattendance rates and ICU admissions among patients discharged from ED with low 4C mortality and deterioration scores.

An important drawback of both scores is that their use may be impractical in low and middle-income countries (LMICs). A recent postmortem surveillance study suggests that COVID-19 rates may have been significantly under-reported in Africa due to poor access to testing.^26^ The 4C scores are only useful after a diagnosis of COVID-19 is confirmed. However, with restricted access to SARS-CoV-2 antigen tests in the community and hospital settings, diagnosis is often made on clinical grounds alone. It can be difficult to distinguish COVID-19 from decompensated heart failure and bacterial pneumonia; this confers a risk of misdiagnosis and inappropriate treatment and management based on irrelevant prognostic scores.

Restricted access to ancillary diagnostic facilities may make it challenging to identify early signs of deterioration or determine prognosis in COVID-19 even where it is possible to establish a diagnosis. In rural LMIC settings, poor access to blood tests and X-ray facilities will make it impossible to calculate the 4C scores. This serves as an urgent reminder of the importance of health systems strengthening in remote LMIC settings, but even with sustained investment and political will it will take years to improve diagnostic capabilities and train local staff. As such, triage tools based on vital signs alone may be more practical and reproducible in these settings. The utility of routinely used EWSs already validated in LMICs—such as the universal vital assessment score developed in sub-Saharan Africa^27^—should be assessed in COVID-19 cohorts alongside external validation of novel models like the PRIEST score developed in high-income settings.^28^ Simpler univariate scoring systems may also be effective. Among 411 adults admitted to a UK urban teaching hospital with COVID-19, admission oxygen saturation on room air alone was a strong predictor of deterioration and mortality.^23^ Healthcare workers and technicians could be rapidly trained to use pulse oximeters and flag patients with hypoxia to medical staff; this would also support judicious use of precious oxygen therapy.^29^ Unfortunately, oximeters remain scarce in countries such as Ethiopia,^30^ and their mass distribution in LMICs should be a priority as the pandemic evolves.

## Future work

Researchers must reassess novel early warning and prognostic scores in light of growing population immunity to prevailing SARS-CoV-2 strains through prior infection or vaccination, and the emergence of new variants associated with higher mortality.^31^ Most prognostic scores for COVID-19 have a short time horizon; they use vital signs and other prognostic markers measured at an index ED attendance or inpatient admission to predict short-term outcomes such as in-hospital mortality and discharge from hospital. However, with a recent retrospective cohort study demonstrating high rates of multiorgan dysfunction and all-cause mortality in COVID-19 survivors at 140 days after hospital discharge,^32^ we need to develop models capable of predicting long-term survival and adverse consequences. Cox regression analyses, which, unlike standard ROC curve analyses, account for the time taken for an adverse event to occur,^33^ would be well suited to the development of these models.

To date, most researchers have taken a crude approach to developing COVID-19 scoring systems, using data from large populations of hospitalised adults assumed to be homogeneous. While evidence is mixed,^34^ some studies support the existence of distinct disease phenotypes, notably a hyperinflammatory subtype associated with higher risks of next-day escalation to higher level respiratory care and higher rates of ICU admission and mortality.^35^ We may see the emergence of novel scores for specific COVID-19 phenotypes and must balance the tension between any additional discriminative benefits they offer and the extra cognitive load they place on overstretched healthcare professionals.

In high-income settings, technology may help to ease this cognitive load and identify high-risk patients across the hospital as close to real time as possible, to aid resource allocation. Future studies should assess whether integration of scores into electronic health records reduces unwarranted variation in treatment escalation and disease outcomes. Scores could be calculated automatically with electronic alerts notifying clinicians of risk and prompting guideline-based clinical management. This could be used to support safe discharge of low-risk patients from the ED and gold-standard prescribing of remdesivir, dexamethasone and tocilizumab at different points in the disease course. The introduction of similar electronic alerts designed to improve the recognition and management of sepsis at a multisite London hospital Trust has previously been shown to reduce mortality.^5^

Future studies which describe the development and validation of novel prognostic scores for COVID-19 must be transparent about their intended purpose. It is often unclear if a score is designed for routine clinical use; to inform risk stratification in interventional studies or to separate different disease phenotypes in observational studies. Prospective external validation may confirm that a novel score reliably discriminates between stable and deteriorating patients, but if the score is difficult to use or understand, it will not be widely adopted. In the UK, one of the key characteristics of the NEWS2 score is that it provides a universal ‘language for sickness’ which is widely understood by healthcare professionals of different stripes and seniority. Close collaboration between clinicians and statisticians at all stages of the research process should aid the development of robust scores which are clinically relevant, easy to use and align with workflow.

Risk prediction tools such as QCOVID have also been developed for patients in the community, to identify those at high risk of acquiring infection and poor outcomes and inform shielding guidelines.^36^ While they may help clinicians and public health agencies to implement targeted risk mitigation measures, they cannot discriminate between patients who can be managed safely in the community and those who require hospital care after acquiring COVID-19. The prevalidation RECAP-V0 is a promising tool which could help to identify patients in a community setting with suspected or confirmed COVID-19 who require further evaluation in secondary care settings.^37^ Future work must seek to determine whether this and similar scores can support more integrated care across whole healthcare systems. For example, early admission of high-risk patients identified in the community may help to avoid spikes of critically ill patients presenting to ED in extremis and enable more equitable distribution of patients across wider hospital networks. This is particularly important in LMICs, where access to advanced respiratory support and critical care is limited.

## Conclusion

EWSs can support timely recognition of clinical deterioration and escalation to critical care or palliation. There are widespread concerns that existing scores such as NEWS2 may fail to identify the deteriorating patient with COVID-19 as they place a premium on cardiovascular instability rather than respiratory dysfunction. Several research groups have used advanced statistical techniques to develop novel early warning and prognostic scores for patients hospitalised with COVID-19. While many of these scores are at high risk of bias, the 4C mortality and deterioration scores have been externally validated in high-income settings and offer useful insights which can inform clinical care. These scores might be used to optimise resource allocation, support discussions around treatment escalation and inform protocols for safe discharge. Unfortunately, limited access to virological testing and laboratory and imaging facilities may blunt their utility in LMICs, where physiological scores may be more practical. Future work should focus on predicting long-term outcomes in COVID-19, improving user experience and identifying the optimum balance between the extra discrimination afforded by novel scores and their ease of use in everyday clinical practice.
